# Double lingual frenulum: a case report

**DOI:** 10.1186/s13256-020-02440-7

**Published:** 2020-07-26

**Authors:** Reham O. Filfilan, Soulafa A. Almazrooa

**Affiliations:** 1 King Abdulaziz University Faculty of Dentistry, Jeddah, Saudi Arabia; 2 Department of Oral Diagnostic Sciences, King Abdulaziz University Faculty of Dentistry, Jeddah, Saudi Arabia

**Keywords:** Lingual frenum, Lingual frenulum, Double lingual frenum, Lingual frenum anomaly

## Abstract

**Background:**

The lingual frenulum is a mucous membrane fold found underneath the tongue. It helps the tongue to perform its function. There are few anomalies that can affect the lingual frenulum, such as ankyloglossia and absence of the lingual frenulum. We report a case of two lingual frenula to educate practitioners about the presence of such an anomaly.

**Case presentation:**

A 10-year-old healthy Saudi girl came to our dental clinic complaining of “malpositioned frontal teeth.” Upon intraoral examination, two lingual frenula were found connecting the tongue with the floor of the mouth. Intraoral examination revealed no other abnormalities.

**Conclusion:**

Double lingual frenulum is an existing frenulum anomaly that did not affect normal function of our patient. A search of the literature revealed that this may well be the first reported case of such a condition.

## Background

The lingual frenulum is a fold of mucous membrane found underneath the tongue. Most of the time, it extends from the midline of the ventral surface of the tongue to the floor of the mouth but not reaching the tip [1]. It helps the tongue to move and perform its functions in swallowing, feeding, and speech. There are few anomalies that can affect the lingual frenulum and sometimes interfere with function, such as ankyloglossia, where the frenulum is attached near the tip of the tongue, commonly described as “tongue tie,” or absence of the lingual frenulum either sporadically or in some developmental conditions, such as Ehlers-Danlos syndrome [2, 3]. To the best of our knowledge, the literature had not previously reported the possibility of having double lingual frenulum; for that reason, we report this case to educate practitioners about the potential presence of such an anomaly.

## Case presentation

**Fig. 1:**
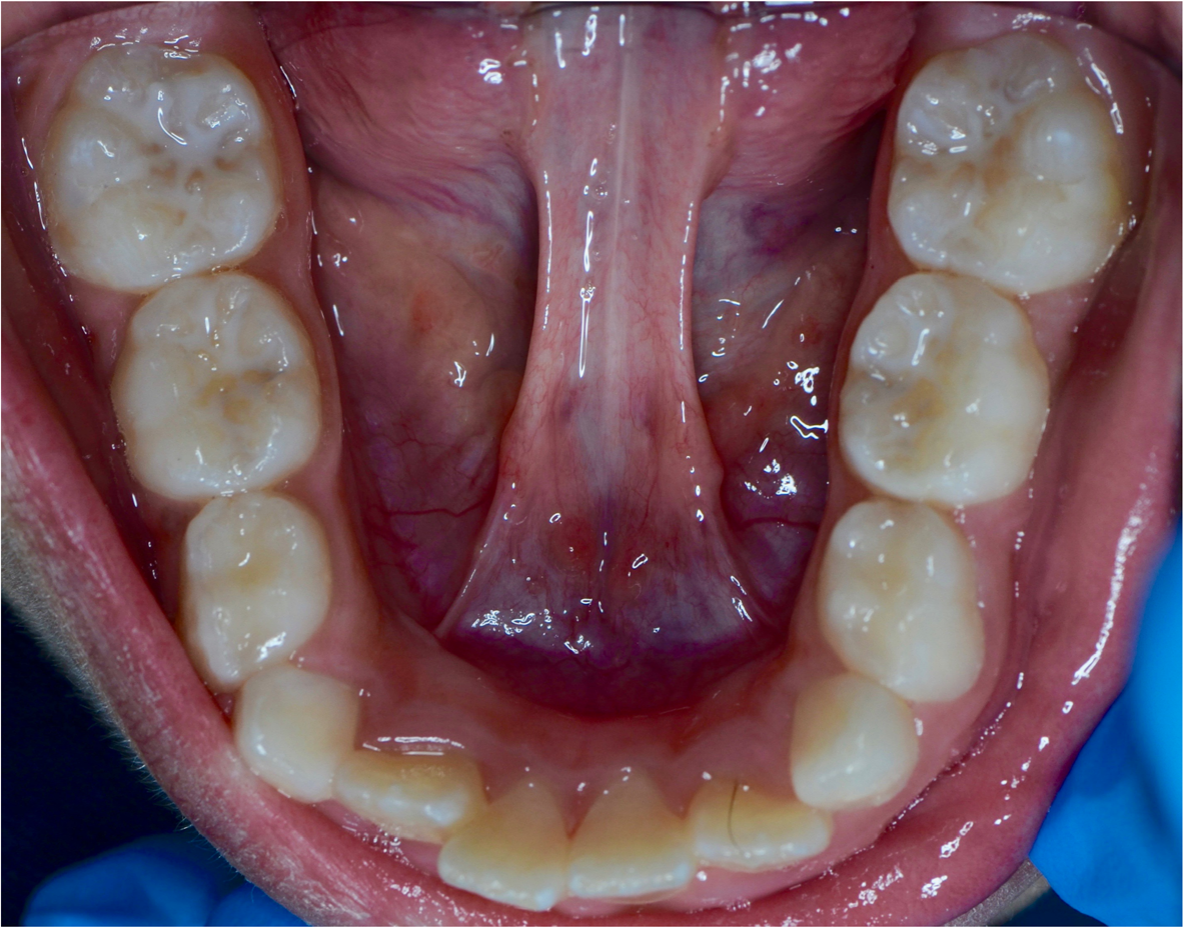
Double lingual frenulum

A 10-year-old Saudi Middle Eastern girl presented to King Abdulaziz University Faculty of Dentistry, Jeddah, Saudi Arabia, complaining of “malpositioned frontal teeth.” The patient’s medical history was insignificant. She had no known allergies or any possible syndrome. Her dental history was significant for multiple restorations. Her family history was insignificant. Her parents reported that their 10-year-old daughter did not pronounce the letters D, T, and Th correctly until the age of 7, and she had not received any previous medical intervention. Extraoral examination showed incompetent lips. Intraoral examination was within normal limits but revealed two lingual frenula with no limitation of tongue function (Fig. 1). The patient faced no challenges. No further management regarding the lingual frenula was required. Her family members were also examined for double lingual frenula as part of the comprehensive assessment process. The patient’s father had a double lingual frenula, and two of her three siblings had no lingual frenulum. None of the family members had any syndromes. A written informed consent was obtained from the patient’s legal guardian for publication of this case report and any accompanying images.

## Discussion

The lingual frenulum guides tongue development and growth *in utero*, during fetal development, and after birth [4]. Alteration in the lingual frenulum can cause abnormal tongue movement leading to alteration in surrounding structure development, such as in orofacial dysmorphosis [5]. Jang *et al.* found that people with skeletal class III malocclusion have a longer lingual frenulum. They also found that the longer the lingual frenulum, the less the degree of mouth opening [6]. After development is completed, the lingual frenulum usually does not extend to the tip of the tongue; it only stabilizes the tongue without causing any interference to tip movement [1].

Walker *et al.* found that the average length from the tongue tip to the insertion of the lingual frenulum in the base of the tongue is between 9 and 10 mm in newborns [7]. The shorter the distance (ankyloglossia), the more problems will be associated, such as difficulties in breastfeeding [7, 8], difficulties in newborn sucking [8], speech impediments [9], teeth malocclusion, maxillary constriction, anterior open bite, spacing in the lower incisors [10], obstructive sleep apnea [5], poor oral hygiene, and being embarrassed by peers [11].

The prevalence of ankyloglossia is 4.2–10.7% [11]. Yet, until now, diagnosing ankyloglossia has been considered difficult due to lack of a standard test. The most common measurements of ankyloglossia were provided by Kotlow and Ruffoli [12, 13]. Kotlow classified the ankyloglossia according to the length of free tongue, from the tongue tip to the insertion of the lingual frenulum in the ventral surface of the tongue [12]. Ruffoli *et al.* classified the ankyloglossia by measuring the maximum opening of the mouth with the tip of the tongue touching the palatal papilla [13]. Other recent studies, such as one by Segal *et al.*, listed the criteria used to diagnose ankyloglossia in studies from 1982 to 2005, but none of the studies assessed internal and external validity [11]. Ingram *et al.* used a Bristol Tongue Assessment Tool as a simple indication for the severity of ankyloglossia but did not assess what level of severity would benefit from the treatment [14]. Yoon *et al.* set a functional tongue range of motion ratio grading scale to define ankyloglossia and the need for surgical treatment, and further studies in this area are needed [4]. Brandão *et al.* used the Neonatal Tongue Screening Test for detecting ankyloglossia, but it was neither reliable nor valid [15]. Ankyloglossia is usually treated by simple frenectomy, cutting of the lingual frenulum. Studies revealed a positive effect on breastfeeding [16–19], but no other studies reported its effect on other tongue-tie problems.

Another known anomaly is absence of the lingual frenulum. It can be seen sporadically or associated with other conditions [3]. It is commonly seen in patients with Ehlers-Danlos syndrome. Literature has suggested that absence of the lingual frenulum can be a simple method of early diagnosis of Ehlers-Danlos syndrome [2, 20]. One more variation reported is posterior lingual frenulum. Martinelli *et al.* stated that, to differentiate between posterior lingual frenulum and absence of lingual frenulum, elevating and pushing the tongue back is required [21]. In our patient’s case, the double lingual frenula required no diagnostic tools other than proper clinical examination, and no intervention was needed, because both frenula did not extend to the tip of the tongue or interfered with the tongue function.

The literature shows few case reports and variations in the labial frenum, such as frenum with a nodule, double frenum, multiple frenula, and high frenum attachment, but no similar literature was found regarding the lingual frenum, except for ankyloglossia, the absence of lingual frenum, and posterior lingual frenum [22–24].

Some syndromes are characterized by supernumerary frenula, such as orofacial-digital syndrome, Pallister-Hall syndrome, and Opitz trigonocephaly C syndrome [22]. However, no literature has ever reported specifically double lingual frenula in any of these syndromes. In addition, our patient did not report any suspicious features of any of these syndromes.

The cause of lingual frenulum variation, including ankyloglossia, absence of lingual frenulum, and posterior lingual frenulum, is unknown, so this is also the case for the double lingual frenulum in our patient.

## Conclusion

Double lingual frenula can be considered a variation from normal. In our patient’s case, it did not affect the tongue functions, so no intervention was needed. Further investigations of a larger population with double lingual frenula are needed to measure the correlation between double lingual frenula and difficulties in breastfeeding, speech impediments, malocclusion, maxillary constriction, and obstructive sleep apnea.

## Data Availability

Case report data and patient's consent form are available.
